# The hOGG1 Ser326Cys polymorphism and prostate cancer risk: a meta-analysis of 2584 cases and 3234 controls

**DOI:** 10.1186/1471-2407-11-391

**Published:** 2011-09-13

**Authors:** Hongtuan Zhang, Yong Xu, Zhihong Zhang, Liang Li

**Affiliations:** 1 Department of Urology, Second Hospital of Tianjin Medical University, Tianjin Institute of Urology, 23 Pingjiang Road, Tianjin, 300211, China; 2 Department of Epidemiology, Second Hospital of Tianjin Medical University, 23 Pingjiang Road, Tianjin, China

## Abstract

**Background:**

Genetic polymorphism of human 8-oxoguanine glycosylase 1 (hOGG1) Ser326Cys (rs1052133) has been implicated to alter the risk of prostate cancer, but the results are controversial.

**Methods:**

Two investigators independently searched the Medline, and Cochrane Library up to June 7, 2011. Summary odds ratios (OR) and 95% confidence interval (CI) for Ser326Cys polymorphism and prostate cancer were calculated. Statistical analysis was performed with the software program Review Manage, version 5.0 and Stata 10.0.

**Results:**

A total of 8 independent studies, including 2584 cases and 3234 controls, were identified. Our analysis suggested that Ser326Cys was not associated with prostate cancer risk in overall population. In the subgroup analysis, we detected the significant association between Ser326Cys polymorphism and decreased prostate risk in mixed population under additive model (OR = 0.67, 95% CI = 0.50-0.90, P = 0.007), recessive model (OR = 0.68, 95% CI = 0.51-0.91, P = 0.008), and Cys allele versus Ser allele (OR = 0.88, 95% CI = 0.78-0.98, P = 0.02). Subanalysis on Caucasian subjects demonstrated that Ser326Cys was not associated with prostate cancer risk.

**Conclusion:**

This meta-analysis showed the evidence that hOGG1 Ser326Cys polymorphism was associated with a decreased risk of prostate cancer development in mixed populations.

## Background

Prostate cancer (PCa) is one of the most common malignant diseases among men. The mechanism of its carcinogenesis, like other cancers, still remains unclear. Multiple environmental and lifestyle factors may increase the risk of PCa, including tobacco use, and a diet poor in fresh fruits and vegetables. However, not all of those who have been exposed to the risk factors will develop PCa, suggesting the inter-individual differences in susceptibility. DNA repair pathways play a critical role in maintaining the genomic integrity in general and specialized functions of cells as well as in the prevention of carcinogenesis, and therefore variations in these genes may lead to higher susceptibility to PCa.

8-Oxodeoxyguanosine, the most abundant lesion generated by oxidative stress from the environment and normal cellular metabolism, is highly mutagenic resulting in GC to TA transversion [1,2]. hOGG1 gene located on chromosome 3 encodes a DNA glycosylase/apurinic-apyrimidinic lyase that catalyzes the excision and removal of 8-hydroy-2-deoxyguanine adducts [3].

In the past years, the hOGG1 Ser326Cys polymorphism has attracted widespread attention. hOGG1 is abundantly expressed in prostate tissue, the functional consequences of the hOGG1 Ser326Cys polymorphism are strongly debated [4]. One study showed that the hOGG1 326Ser enzyme have higher activity than the 326Cys variant enzyme [5]. Another study data suggested that both 326Ser and 326Cys variant enzymes are functional and do not exhibit significant differences in repair activities [6]. The other study indicated that capacity to repair oxidative DNA damage was significantly decreased in individuals with 326Cys/Cys compared with Ser/Ser and Ser/Cys genotypes [7]. Several eligible case-control studies were performed to identify the association of Ser326Cys polymorphism with PCa risk [8-15]. However, the results remain inconclusive and inconsistent.

To date, no meta-analysis has been conducted to investigate the association of Ser326Cys polymorphism of hOGG1 gene and PCa. Hence, a meta-analysis based on a total of 8 independent studies was performed, which may provide the evidence for association of hOGG1 Ser326Cys polymorphism with PCa susceptibility.

## Methods

### Publication search

We searched the articles using the terms "hOGG1" or "OGG1", "polymorphism" or "variation", "prostate" and "cancer" in Medline and Cochrane Library, and all eligible studies were detected before June 7, 2011. We evaluated all associated publications to retrieve the most eligible literatures. Their reference lists were hand-searched to find other relevant publications. Of the studies with overlapping data published by the same investigators, only the most recent or complete study was included in this meta-analysis.

### Inclusion and exclusion criteria

The following inclusion criteria were used to select literatures for the meta-analysis: (1) Only the case-control studies were considered; (2) The paper should clearly describe PCa diagnoses and the sources of cases and controls; (3) The authors must offer the size of the sample, OR and their 95% CI or the information that can help infer the results in the papers. The exclusion criteria were: (1) none-case-control studies; (2) control population including malignant tumor patients; and (3) duplicated publications.

### Data Extraction

Two investigators reviewed and extracted information from all eligible publications independently, according to the inclusion and exclusion criteria listed above. An agreement was reached by discussion between the two reviewers whenever there was a conflict. The following items were collected from each study: first author's surname, year of publication, statistical data, ethnicity, total number of cases and controls as well as numbers of cases and controls with Ser/Ser, Ser/Cys, and Cys/Cys genotypes, respectively. Different descents were categorized as Caucasian, Asian, African and Mixed, which included more than one ethnic descent.

### Statistical analysis

The effect measure of choice was OR with its corresponding 95% CI. The significance of the summary OR was determined with a Z-test and P < 0.05 was considered as statistically significant. In our study, two models of meta-analysis were applied for dichotomous outcomes: the fixed-effects model and the random-effects model. The fixed-effects model assumes that studies are sampled from populations with the same effect size, making an adjustment to the study weights according to the in-study variance. The random-effects model assumes that studies are taken from populations with varying effect sizes, calculating the study weights both from in-study and between-study variances, considering the extent of variation, or heterogeneity. Heterogeneity assumption was checked by Q-test. P ≥ 0.10 for the Q-test indicated lack of heterogeneity among the studies. Either a random-effects model or fixed-effects model was used to calculate pooled effect estimates in the presence or absence of heterogeneity [16,17], respectively. To explore the effect of heterogeneity among the studies on the conclusions of this meta-analysis, subgroup analyses were performed by ethnicity.

Firstly, we examined Ser326Cys genotypes using additive (Cys/Cys vs Ser/Ser), recessive (Cys/Cys vs Ser/Cys + Ser/Ser) and dominant (Cys/Cys + Ser/Cys vs Ser/Ser) genetic models. Then, the comparison of Cys allele with Ser allele (allelic model) was examined. An asymmetric plot indicates a possible publication bias. The symmetry of the funnel plot was further evaluated by Egger's linear regression test. The significance of the intercept was determined by the t-test suggested by Egger (P < 0.05 was considered representative of statistically significant publication bias). All statistical tests were performed with Review Manage, version 5.0 and Stata 10.0 using two-sided P-values.

## Results

### Eligible Studies

8 publications on hOGG1 Ser326Cys genotypes and PCa were identified through literature search and selection based on the inclusion and exclusion criteria [8-15]. 8 independent studies consisted of 3 Caucasian, 1 Asian, 1 African and 3 mixed populations. In total, 2584 PCa cases and 3234 controls were included in the meta-analysis. The selected study characteristics were summarized in Table 1.

**Table 1:** Main characteristics of studies included in this meta-analysis.

Investigator [reference]	Year	Ethnicity	Cases			Controls		
			
			Ser/Ser	Ser/Cys	Cys/Cys	Ser/Ser	Ser/Cys	Cys/Cys
Yun et al. [8]	2011	Asian	54	119	93	68	131	67
Zhang et al. [9]	2010	Mixed	126	61	4	118	71	7
Lavender et al. [10]	2010	African	132	58	4	452	173	21
Dhillon et al. [11]	2009	Caucasian	38	57	21	69	50	12
Nock et al. [12]	2006	Mixed	280	135	24	305	142	31
Nam et al. [13]	2005	Mixed	593	350	53	617	386	89
Chen et al. [14]	2003	Caucasian	49	29	6	185	63	3
Xu et al. [15]	2002	Caucasian	182	106	10	96	63	15

### Meta-analyses and Evaluation of Heterogeneity and Publication bias

**Figure 1:**
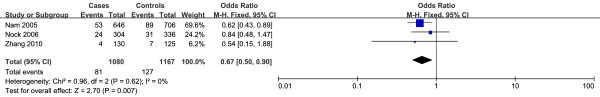
**Forest plots for hOGG1 Ser326Cys polymorphism and risk of Prostate cancer in mixed population (Cys/Cys versus Ser/Ser)**. The squares and horizontal lines correspond to the study specific OR and 95% CI. The area of the squares reflects the weight (inverse of the variance). The diamond represents the summary OR and 95% CI.

**Figure 2:**
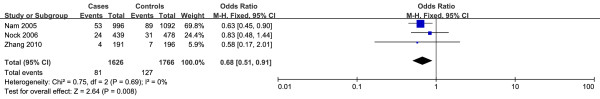
**Forest plots for hOGG1 Ser326Cys polymorphism and risk of Prostate cancer in mixed population (Cys/Cys versus Ser/Cys + Ser/Ser)**. The squares and horizontal lines correspond to the study specific OR and 95% CI. The area of the squares reflects the weight (inverse of the variance). The diamond represents the summary OR and 95% CI.

**Figure 3:**
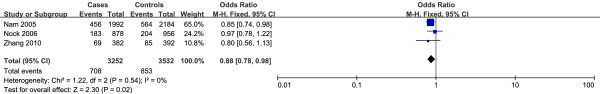
**Forest plots for hOGG1 Ser326Cys polymorphism and risk of Prostate cancer in mixed population (Cys allele versus Ser allele)**. The squares and horizontal lines correspond to the study specific OR and 95% CI. The area of the squares reflects the weight (inverse of the variance). The diamond represents the summary OR and 95% CI.

The meta-analysis for association of Ser326Cys polymorphism with PCa in overall population included 8 independent studies with a total of 2584 cases and 3234 controls. The Q-test of heterogeneity was significant and we conducted analyses using random effect models. There was no statistically significant difference in Pca risk between the patients with Cys/Cys genotype and those with Ser/Ser genotype (OR 1.06, 95% CI = 0.61-1.87, P = 0.83). Similarly, no significant associations were found in the recessive model comparison (OR 1.00, 95% CI = 0.61-1.63, P = 0.99) and dominant model comparison (OR 1.12, 95% CI = 0.89-1.40, P = 0.33). In addition, we did not detect the association between Ser326Cys polymorphism and PCa when examining the contrast of Cys versus Ser (OR 1.10, 95% CI = 0.88-1.37; P = 0.41). In the stratified analysis by ethnicity, significant between-study heterogeneity was detected in all the comparisons in Caucasians, but not in mixed population. For mixed population, there was significant association between Ser326Cys polymorphism and decreased PCa for additive model comparison (OR = 0.67, 95% CI = 0.50-0.90, P = 0.007; Figure 1), recessive model comparison (OR = 0.68, 95% CI = 0.51-0.91, P = 0.008; Figure 2), and Cys allele versus Ser allele comparison (OR = 0.88, 95% CI = 0.78-0.98, P = 0.02; Figure 3), except for the dominant model comparison (OR = 0.90, 95% CI = 0.78-1.03, P = 0.13, data not shown in Figure). We did not find any associations between Ser326Cys polymorphism and PCa risk in Caucasians. Begg's funnel plot and Egger's test were performed to assess the publication bias. The results did not show any evidence of publication bias in all the comparisons. The detailed data were shown in Table 2.

**Table 2:** Meta-Analysis of hOGG1 Ser326Cys Polymorphism and PCa

Ser326Cys polymorphism Genetic model (No. of studies)	Sample size		Analysis	Test of association		P value for heterogeneity	P value for Egger's test
					
	Case	Control	model	OR (95% CI)	P		
Overall (8)							
Cys/Cys vs Ser/Ser	1669	2155	R	1.06 (0.61-1.87)	0.83	< 0.00001	0.522
Cys/Cys vs Ser/Cys + Ser/Ser	2584	3234	R	1.00 (0.61-1.63)	0.99	< 0.0001	0.823
Cys/Cys + Ser/Cys vs Ser/Ser	2584	3234	R	1.12 (0.89-1.40)	0.33	0.001	0.082
Cys vs Ser	5168	6468	R	1.10 (0.88-1.37)	0.41	< 0.00001	0.158
Caucasian (3)							
Cys/Cys vs Ser/Ser	306	292	R	1.92 (0.32-11.43)	0.47	< 0.0001	0.661
Cys/Cys vs Ser/Cys + Ser/Ser	498	556	R	1.60 (0.34-7.48)	0.55	0.0004	0.668
Cys/Cys + Ser/Cys vs Ser/Ser	498	556	R	1.50 (0.73-3.08)	0.27	0.0009	0.057
Cys vs Ser	996	1112	R	1.40 (0.70-2.78)	0.34	< 0.00001	0.251
Mixed (3)							
Cys/Cys vs Ser/Ser	1080	1167	F	0.67 (0.50-0.90)	0.007	0.62	0.964
Cys/Cys vs Ser/Cys + Ser/Ser	1626	1766	F	0.68 (0.51-0.91)	0.008	0.69	0.920
Cys/Cys + Ser/Cys vs Ser/Ser	1626	1766	F	0.90 (0.78-1.03)	0.13	0.58	0.875
Cys vs Ser	3252	3532	F	0.88 (0.78-0.98)	0.02	0.54	0.982

PCa = prostate cancer; OR = odds ratio; CI = confidence interval; vs = versus; R = random effect model; F = fixed effect model.

## Discussion

Several DNA repair pathways are involved in the maintenance of genetic stability. The hOGG1 gene is involved in base excision repair (BER) of DNA repair pathways. DNA damage generated by different carcinogenic agents or inflammatory process is repaired mostly by BER. Common polymorphisms in DNA repair genes may alter protein function and an individual's capacity to repair damaged DNA. Deficits in repair capacity may lead to genetic instability and carcinogenesis [18,19]. So allelic variants in the key gene involved in DNA repair process, hOGG1, may confer an increased risk for PCa development.

Since the identification of hOGG1 Ser326Cys polymorphism, a number of studies have investigated the genetic effect of this polymorphism on PCa susceptibility, but the results are inconclusive. As a powerful statistical method, meta-analysis can provide a quantitative approach for pooling the results of different researches on the same topic, and for estimating and explaining their diversity [20,21]. This led us to undertake the present meta-analysis, which could quantitify all the available data and might help us to distinguish the true from the false, to explore a robust estimate of the effect of this polymorphism on PCa. To the best of our knowledge, it is the first systematic review that has investigated the association of hOGG1 Ser326Cys polymorphism and PCa. In present meta-analysis, no evidence has shown any associations between Ser326Cys polymorphism and PCa susceptibility in overall population.

The results of several studies have suggested that single nucleotide polymorphisms may determine the differences in the risk of PCa between ethnic groups [22,23]. Ethnic differences in the incidence of PCa are well established. So subanalysis on different ethnicity was performed. Our data suggested that hOGG1 Ser326Cys polymorphism was associated with a statistically significant decrease in PCa risk in mixed population. However, no significant association was detected in Caucasians. This indicates a possible role of ethnic differences in genetic backgrounds and the environment they lived in. Moreover, the discrepancy might be due to chance because studies with small sample sizes may be underpowered to detect a slight effect or may have generated a fluctuated risk estimate. Therefore, the results of this study should be interpreted with caution.

In the present study, statistically significant between-study heterogeneity of genotype effect was detected in all different genetic models when all the eligible studies were pooled into the meta-analysis. After subgroup analyses by ethnicity, significant heterogeneity was only detected in Caucasians. The heterogeneity was effectively removed in mixed subgroup. Significant between-study heterogeneity was detected in overall and Caucasian population and we conducted analyses using random effect models. Then we got a wider confidence interval and a larger P-value which may be distorting the meta-analysis. The data showed that no obvious publication bias existed in our meta-analysis.

Some limitations of this meta-analysis should be acknowledged. First, controls were not uniformly defined, so selection bias may occur and they may not be representative of the general population. Second, the number of cases and controls in the included studies was relatively low. Third, our result was based on unadjusted estimates, while a more precise analysis should be conducted adjusted by other factors like smoking, drinking status and environmental factors. Fourth, in the subgroup analyses by ethnicity, relatively limited study number and incomplete information for mixed ethnicities made it impossible to perform ethnic subgroup analysis of Africans and Asians. Thus, additional studies are warranted to evaluate the effect of this functional polymorphism on PCa risk in different ethnicities, especially in Africans and Asians. In addition, our analysis did not consider the possibility of gene-gene or SNP-SNP interactions or the possibility of linkage disequilibrium between polymorphisms. Therefore, larger and well-designed studies are needed to further evaluate the association between hOGG1 polymorphism and PCa risk.

## Conclusions

Our meta-analysis evaluated the association between Ser326Cys polymorphism and PCa risk and revealed that hOGG1 polymorphism Ser326Cys was associated with a statistically significant decrease in PCa risk in mixed population. Due to limitations showed above in this analysis, it is critical that larger and well-designed multicenter studies are needed to confirm our results.

## List of abbreviations

hOGG1: human 8-oxoguanine glycosylase 1; PCa: prostate cancer; BER: base excision repair; OR: odds ratio; CI: confidence interval; vs: versus; R: random effect model; F: fixed effect model.

## Competing interests

The authors declare that they have no competing interests.

## Authors' contributions

ZH carried out the publication search, participated in data analysis and drafted the manuscript. XY carried out publication search, and revised the manuscript. ZZ participated in the publication search and helped to draft the manuscript. LL participated performed the statistical analysis. All authors read and approved the final manuscript.

## Pre-publication history

The pre-publication history for this paper can be accessed here:

http://www.biomedcentral.com/1471-2407/11/391/prepub
